# Large-scale prediction of activity cliffs using machine and deep learning methods of increasing complexity

**DOI:** 10.1186/s13321-022-00676-7

**Published:** 2023-01-07

**Authors:** Shunsuke Tamura, Tomoyuki Miyao, Jürgen Bajorath

**Affiliations:** 1 Department of Life Science Informatics, B-IT, LIMES Program Unit Chemical Biology and Medicinal Chemistry, Rheinische Friedrich-Wilhelms-Universität, Friedrich-Hirzebruch-Allee 5/6, 53115 Bonn, Germany; 2 Graduate School of Science and Technology, Nara Institute of Science and Technology, 8916-5 Takayama-cho, Ikoma, Nara 630-0192 Japan; 3 Data Science Center, Nara Institute of Science and Technology, 8916-5 Takayama-cho, Ikoma, Nara 630-0192 Japan

**Keywords:** Activity cliff, Machine learning, Deep learning, Compound pair-based prediction, Large-scale analysis

## Abstract

Activity cliffs (AC) are formed by pairs of structural analogues that are active against the same target but have a large difference in potency. While much of our knowledge about ACs has originated from the analysis and comparison of compounds and activity data, several studies have reported AC predictions over the past decade. Different from typical compound classification tasks, AC predictions must be carried out at the level of compound pairs representing ACs or nonACs. Most AC predictions reported so far have focused on individual methods or comparisons of two or three approaches and only investigated a few compound activity classes (from 2 to 10). Although promising prediction accuracy has been reported in most cases, different system set-ups, AC definitions, methods, and calculation conditions were used, precluding direct comparisons of these studies. Therefore, we have carried out a large-scale AC prediction campaign across 100 activity classes comparing machine learning methods of greatly varying complexity, ranging from pair-based nearest neighbor classifiers and decision tree or kernel methods to deep neural networks. The results of our systematic predictions revealed the level of accuracy that can be expected for AC predictions across many different compound classes. In addition, prediction accuracy did not scale with methodological complexity but was significantly influenced by memorization of compounds shared by different ACs or nonACs. In many instances, limited training data were sufficient for building accurate models using different methods and there was no detectable advantage of deep learning over simpler approaches for AC prediction. On a global scale, support vector machine models performed best, by only small margins compared to others including simple nearest neighbor classifiers.

**Graphical Abstract:**

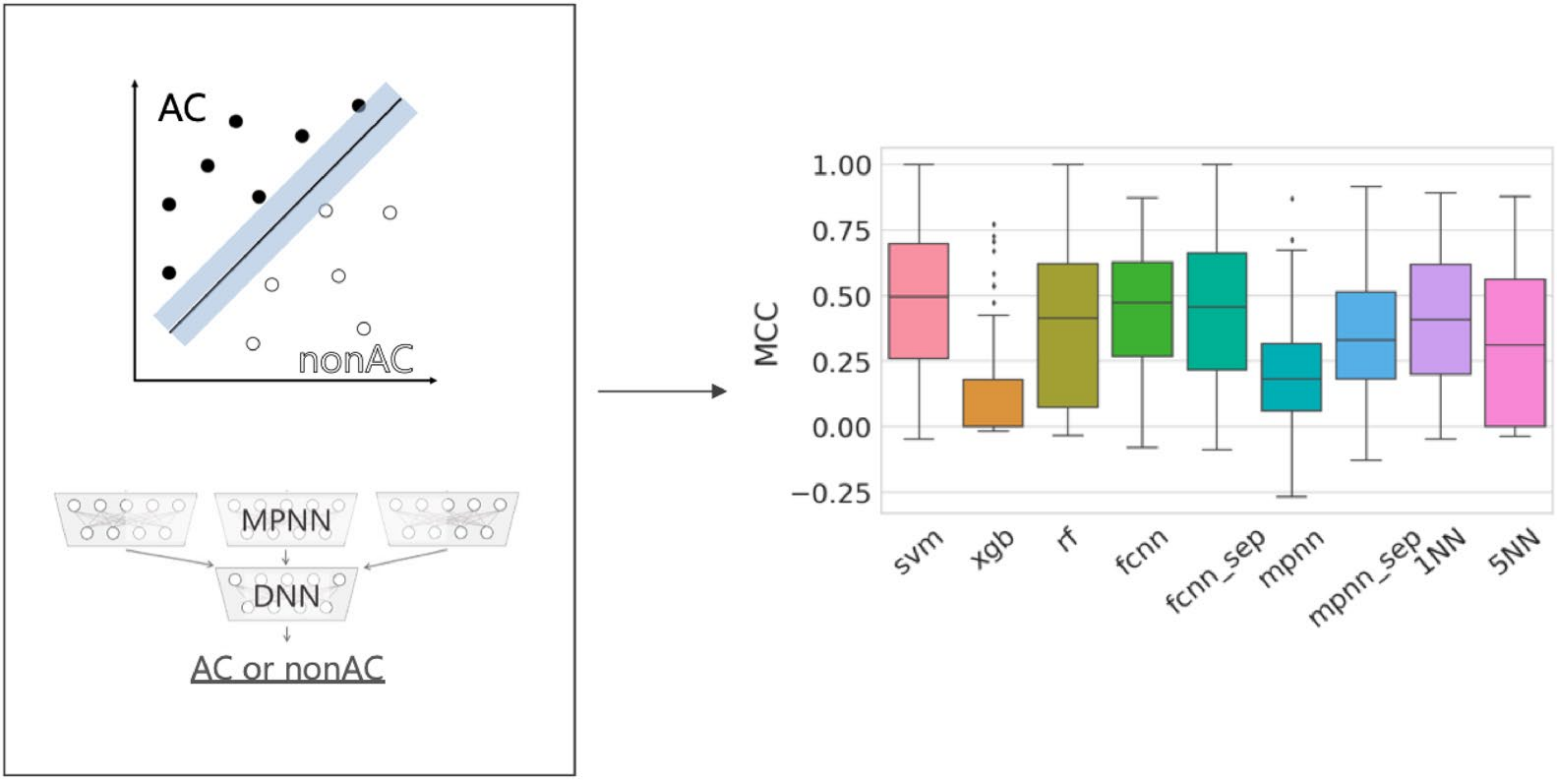

**Supplementary Information:**

The online version contains supplementary material available at 10.1186/s13321-022-00676-7.

## Introduction

Activity cliffs (ACs) were originally defined as structural similar active compounds with large differences in potency  (against the same target), presenting major problems for standard quantitative structure–activity relationship (QSAR) predictions [1]. ACs are of high relevance for medicinal chemistry, given that they capture small chemical modifications with large consequences for specific biological activities [2]. As such, ACs represent extreme examples of SAR discontinuity, which is encountered during compound optimization and might (or might not) be desirable, depending on the development stage of compound series [2]. In medicinal chemistry, ACs are best rationalized as structural analogues (belonging to the same series) having large potency differences. However, for AC definition and assessment, a variety of molecular similarity and potency difference criteria have been introduced [2] and the AC concept has been further refined over time, both from a medicinal chemistry and chemoinformatics perspective [3].

While ACs were systematically explored on the basis of compound activity data analysis [2], which yielded most of our current insights into ACs and their distribution across different compound classes [2, 3], various attempts have also been made to computationally predict ACs [3]. Compared to predictions of compound activity (or other molecular properties) using QSAR modeling including machine learning (ML), AC predictions were principally challenging because they needed to focus on compound pairs, rather than individual molecules, which required methodological adjustments and extensions. First attempts to predict individual AC compounds (i.e., compounds participating in the formation of an AC) or complete ACs were made a decade ago using random forest (RF) and support vector machine (SVM), respectively [4, 5]. In these studies, SVM predictions using matched molecular pair (MMP) representations of ACs and especially designed MMP kernels to facilitate predictions at the level of compound pairs yielded surprisingly high prediction accuracy (80–90%). An MMP is defined as a pair of compounds with a chemical change (exchange of a substituent) at a single site. Therefore, the SVM-MMP formalism was also applied in subsequent studies to further explore and refine AC predictions [6, 7]. As a simpler alternative to the use of MMP kernels, the condensed graph of reaction (CGR) formalism was also applied to represent MMPs for AC predictions using different QSAR/ML methods, reaching an accuracy overall comparable to SVM [8]. For representing individual compounds or MMPs, standard fingerprint descriptors from chemoinformatics, for the most part bit string representations of chemical structure, were used in these studies. Following a different approach, ACs were also predicted on the basis of target-bound compound conformations and three-dimensional (3D) binding mode similarity measures [9], yielding lower accuracy than SVM modeling. Recently, deep learning (DL) has been applied to predict ACs from MMP images using convolutional neural networks [10, 11] or from molecular graphs involving representation learning with graph neural networks (GNNs) [12]. These DL approaches to AC prediction reached similarly high prediction accuracy as earlier ML studies (for example, with area under the receiver-operating characteristic curve (AUC) values greater 0.9). Furthermore, a transformer-based chemical language model has recently been introduced to bridge between AC prediction and the design of new AC compounds [13], hence adding a new dimension to predictive modeling. This model also achieved AC prediction accuracy comparable to (or better than) other state-of-the-art ML models [13]. In addition to classification models for AC prediction, regression models have also been applied to predict the potency of individual AC compounds [14, 15].

All AC prediction efforts reported over time applied a general 100-fold difference in compound potency as a criterion for AC definition, irrespective of the compound classes under investigation. Furthermore, with the exception of 3D AC predictions [9], these studies consistently applied the MMP formalism as a similarity criterion for AC definition and representation. Moreover, all of these studies also had in common that they reported AC predictions only for a limited number of compound activity classes; always fewer than 10 and in some cases –including the DL investigations– only two to four. An activity class is defined as a set of compounds with experimentally confirmed activity against a given target. Since the system set-up, compound classes, and calculation conditions largely varied in the studies, they can also not be rigorously compared.

In this work, we report the first large-scale prediction of ACs over 100 compound activity classes using ML methods of increasing complexity including DL. For each activity class, ACs and nonACs (MMPs not meeting AC potency difference criteria) were identified and classification models were built to systematically distinguish between ACs and nonACs. By design, this study aimed to enable a direct comparison of various methodologies for AC prediction and provide a general assessment of prediction accuracy across many different compound classes. Furthermore, different from earlier studies, ACs were defined and predicted on the basis of statistically significant activity class-dependent potency differences derived from class-specific compound potency distributions, hence further refining the assessment of ACs.

## Methods

### Compound data sets

Compound activity classes were extracted from the ChEMBL database (version 29) [16] based on the following criteria: molecular mass less than 1000 Da, target confidence score of 9, interaction relationship type ‘D’, and availability of a numerically specified potency value. Only *K*_*i*_ or *K*_*d*_ measurements were considered as potency annotations. Each activity class consisted of qualifying compounds with reported activity against an individual target. In addition to the compound-based selection criteria given above, activity classes were required to meet AC analysis criteria, as specified below. A total of 100 activity classes were assembled. Their targets and composition are reported in Additional file 1: Table S1.

### Activity cliff definition

#### Structural similarity criterion

As an intuitive representation of structurally analogous compounds with small chemical modifications, the MMP formalism was applied. An MMP is formed by a pair of compounds that share a common core structure and are distinguished by substituents at a single site. An MMP-based AC, termed MMP-cliff, was defined as an MMP with a large difference in potency between the participating compounds (as further detailed below) [17]. For AC analysis, MMPs were generated with the computationally efficient molecular fragmentation algorithm introduced by Hussain and Rea [18] using a previously reported implementation [19]. For MMP generation, a substituent was permitted to consist of at most 13 non-hydrogen atoms and the core structure was required to be at least twice as large as a substituent. The maximum difference in non-hydrogen atoms between the exchanged substituents was set to eight non-hydrogen atoms [17]. Generated MMPs having a core with less than 10 non-hydrogen atoms were discarded.

#### Activity class-dependent potency difference criteria

Most of the previously reported AC analyses and predictions applied a constant 100-fold difference in potency as a criterion, regardless of the compound classes under study [20]. However, the analysis of compound potency distributions across many activity classes has shown that a 100-fold difference in potency can only serve as an approximate criterion for AC definition [21]. Instead, from class-dependent compound potency distributions, statistically significant potency differences qualifying for ACs were determined as the mean compound potency per class plus two standard deviations, yielding more realistic variable class-dependent potency difference criteria [21], as also applied herein. Furthermore, to balance potency difference-dependent boundary effects in AC prediction, only MMPs with a less than tenfold difference in potency (∆*pK*_*i*_ < 1) were classified as nonACs.

### Compound overlap in matched molecular pairs

Different MMPs from an activity class might share individual compounds. When MMPs are randomly divided into training and test sets, MMPs with compound overlap might appear in both sets, giving rise to high similarity between such training and test instances. Accordingly, compound overlap between training and test MMPs causes a form of “data leakage”, favoring similarity-based detection of MMPs with shared compounds [12]. To address the influence of data leakage phenomena on model performance, we generated different MMP partitions for training and testing in the presence or absence of data leakage. Under “data leakage possibly included” conditions, MMPs from 100 activity classes were randomly divided into training (80%) and test sets (20%). By contrast, under “data leakage excluded” conditions, an advanced cross-validation (AXV) approach was applied [8]. Accordingly, for each activity class, a hold-out set of 20% of the compounds was randomly selected before MMPs were generated for the entire class. If neither compound of an MMP was present in the hold-out set, the MMP was assigned to the training set. If both MMP compounds (forming the MMP) were contained in the hold-out set, the MMP was assigned to the test set. If one of the MMP compounds was present in the hold-out set, the MMP was omitted from training and test sets. For predictions under “data leakage excluded” conditions, 42 activity classes yielding at least 20 ACs were selected to ensure meaningful model derivation and evaluation.

### Molecular representation

#### Fingerprints

Extended connectivity fingerprints with bond diameter 4 (ECFP4) [22] were used to represent MMPs. As a modification, features with bond diameter 1 were omitted to reduce feature sets and emphasize contributions of features with larger bond diameters. Feature identifiers were sorted in ascending order and assigned to fingerprint bits in the same order to prevent feature collision and maximize the number of features contributing to AC prediction. Fingerprints were separately generated for the core and chemical transformation of an MMP. For the transformation, two fingerprints were generated including one recording unique features of the exchanged substituents and another recording common features. Then, the fingerprints for the core, unique features of the substituents, and common features of substituents were concatenated to produce a single MMP fingerprint [7]. Accordingly, the length of the fingerprint depended on each activity class. MMP fingerprint calculations were conducted with in-house Java and Python scripts based on the *OEChem toolkit* [23].

#### Condensed graph of reaction representation

For neural network calculations, MMPs were also represented as a single graph applying the condensed graph of reaction (CGR) approach [8, 24]. The CGR formalism was originally conceived to combine reactant and product graphs based on a superposition of invariant components [24]. The resulting CGR forms a completely connected graph in which each node represents an atom and each edge a bond. In a CGR, the shared core of an MMP and the two exchanged substituents form a pseudo-molecule. Here, the subgraphs representing the substituents of the weakly and highly potent MMP compounds were connected to the core via a single bond and a hypothetical zero-order bond, respectively. The pseudo-molecule representation of MMPs was generated using an in-house Python script and *RDKit* [25].

### Machine learning

Four fingerprint-based ML approaches for AC prediction were applied including SVM, extreme gradient boosting (XGB), RF, and a fully connected neural network (FCNN). In addition, a message passing neural network (MPNN) involving representation learning from graphs was used. For FCNN and MPNN, two distinct models were generated on the basis of different molecular representations (see below). As controls, *k*-nearest neighbor (kNN) calculations including 1NN and 5NN were carried out, in which similarity was evaluated using the MMP kernel described below.

For each activity class and ML method, three independent models were derived with three-fold internal cross-validation to optimize hyperparameters. Model performance was average over three independent trials.

FCNN and MPNN were implemented using *PyTorch* [26] and all other models using *scikit-learn* [27]*.* Hyperparameters of models were optimized using *Optuna* library [28], as reported in Additional file 1: Table S2 (for remaining parameters, default settings were used). For each model, the hyperparameter search with *Optuna* was performed 100 times.

#### Support vector machine

SVM is a supervised learning method that aims to derive a hyperplane separating training instances with different class labels by maximizing the margin from the hyperplane [29]. SVM can attempt nonlinear classification in higher-dimensional feature spaces with the aid of kernel functions. Herein, the MMP kernel [5] was used that represents a product of two individual Tanimoto kernels [30] for determining core and substituent similarity, respectively. The parameter ‘class_weight’ was set to ‘balanced’. The hyperparameter C was selected using grid search from the value range [$\mathrm{log}\left(-2\right),$ $\mathrm{log}2]$ divided into 10 equal intervals.

#### Random forest

RF is a supervised ML method based upon an ensemble of decision trees generated from randomly chosen training instances using bootstrapping [31]. Class labels of test instances are predicted by a majority vote over individual decision trees. The parameter ‘class_weight’ was set to ‘balanced’.

#### Extreme gradient boosting

XGB also employs an ensemble of decision trees iteratively generated using gradient boosting [32] such that each decision tree minimized the residual error from a previous model. XGB is a computationally efficient extension of gradient boosting achieved by parallelizing decision tree construction.

#### Neural networks

##### Fully connected neural network

A FCNN consists of a series of connected perceptrons stored in several layers. Each perceptron receives signals from the previous layer that are transformed into scalar values using an activation function. In this study, two distinct FCNNs were implemented using different input representations including a single MMP fingerprint (FCNN) or the three separate core and substituent fingerprint components (FCNN_sep). MMP fingerprints were converted into probabilities of AC formation. The number of nodes in hidden layers was monotonically reduced. In FCNN_sep, the individual fingerprint components were submitted to several hidden layers and the output fingerprints were concatenated into a single vector, which was sent to subsequent hidden layers and transformed into the probability of AC formation using softmax layer. The number of nodes in hidden layers for both individual substructures and concatenated feature vectors was also monotonically reduced. The Rectified Linear Unit (ReLU) [33] was used as activation function, except for the final layer. Binary cross-entropy with balance factor weighted by the ratio of  negative to positive samples was used as loss function for the Adam optimizer [34]. The learning rate was facilitated by the optim.lr_scheduler.StepLR in PyTorch. For the scheduler, the parameter gamma was set to 0, while the step size was an optimized hyperparameter. The batch size was set to 128 if the number of MMPs in a training set was greater than 128; otherwise, it was set to the size of the training set. Training steps were performed for 50 epochs during the hyperparameter search and for 100 epochs during fitting using preferred parameters.

##### Message passing neural network

MPNN is a graph neural network approach converting an input molecular graph into a feature vector. During MPNN training, a feature vector of each atom is iteratively merged with information from its neighboring atoms and bonds to minimize the loss function. The initial features for each atom and bond are listed in Additional file 1: Table S3. The transformed feature vectors of each atom are merged into single vector submitted to a fully-connected neural network with several hidden layer producing an output probability. Herein, a previously implemented MPNN architecture [35] originally proposed by Tang et al. [36] was used. In analogy to FCNN and FCNN_sep, two distinct MPNNs were generated based on a single CGR as input (MPNN) or three separate subgraphs representing the MMP core and substituents, respectively (MPNN_sep). In the latter case, feature vectors for each substructure were individually calculated and then concatenated into single vector as input for the fully-connected neural network. Activation function, loss function, optimizer, scheduler of optimizer, batch size, epochs, and number of hyperparameter search calculations were set as reported for FCNN.

### Performance measures

To evaluate the performance of the different models, balanced accuracy (BA) [37], recall, precision, and Matthew’s correlation coefficient (MCC) [38] were determined.

## Results and discussion

### Study concept

Previous studies predicting ACs predominantly focused on individual ML methods and generally investigated only small numbers of activity classes. By contrast, our current investigation was designed to compare AC predictions on a large scale classes using ML methods of varying complexity, ranging from nearest neighbor calculations to deep neural networks. Accordingly, our study aimed to arrive at a comprehensive assessment of AC predictions, taking class-specific potency difference thresholds for AC formation into account, and provide general insights into performance differences between methods of varying computational complexity and requirements.

### Global performance comparison

**Fig. 1:**
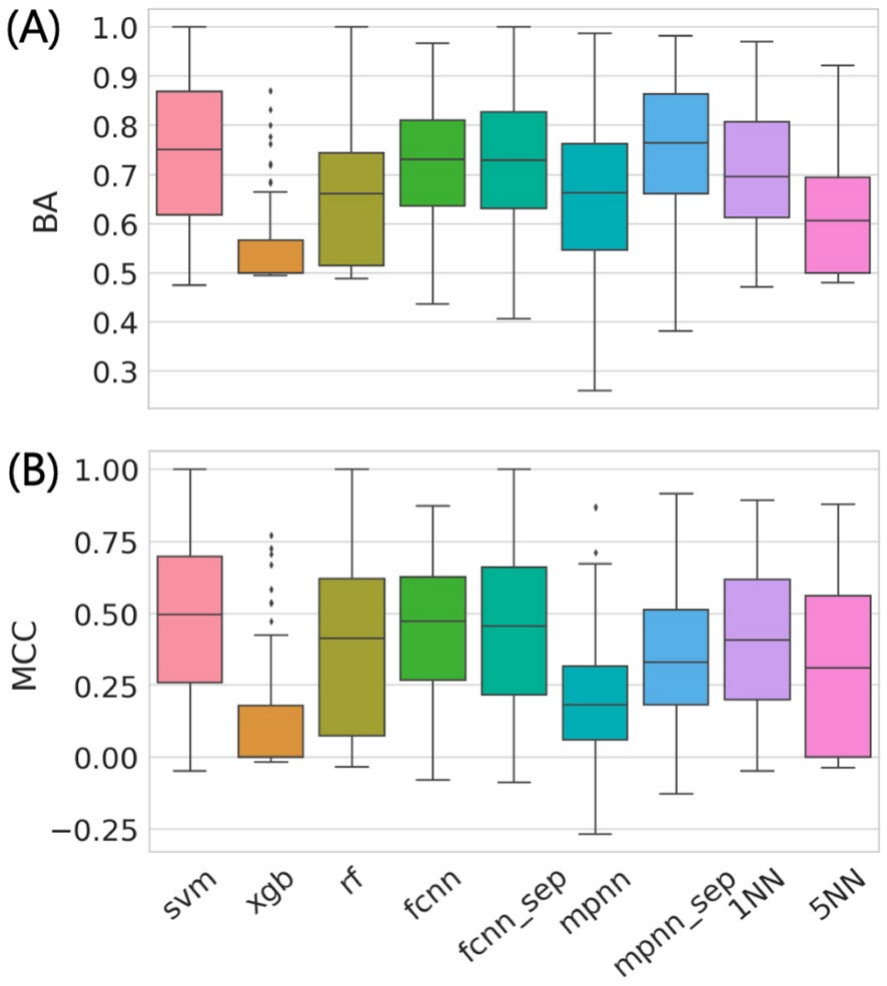
Global prediction accuracy. Boxplots report the distribution of AC prediction accuracy for nine ML approaches across 100 activity classes on the basis of **A** BA and **B** MCC values. The models were built based on randomly selected training and test sets (that is, under “data-leakage possibly included” conditions; see Methods section). In a boxplot, a value distribution is represented by its maximum (upper whisker), upper quartile (upper boundary of the box), median (horizontal line), lower quartile (lower boundary of the box) and its minimum (lower whisker). Individual values representing statistical outliers are shown as black dots

The accuracy of AC predictions across 100 different activity classes using nine different methods is summarized in Fig. 1. Both on the basis of BA and MCC performance measures, most models were predictive, with median BA values of ~ 0.7 or greater and positive median MCC values of up to ~ 0.5. Interestingly, decision tree methods including RF and XGB as well as MPNN displayed overall lowest performance, with XGB approaching random prediction accuracy on the basis of both performance measures. By contrast, SVM, FCNN, and 1NN (but not 5NN) performed comparably well. Notably, the simple 1NN classifier approached the performance level of much more complex ML models, indicating that many ACs were more similar to other ACs than to nonACs (and vice versa); an interesting finding. When similarity was averaged over five nearest neighbors (5NN) prediction accuracy decreased (thus emphasizing closets relationships).

Furthermore, while there were essentially no differences in performance between the FCNN and FCNN_sep model variants, MPNN_sep achieved significantly higher prediction accuracy than MPNN, also slightly exceeding SVM on the basis of BA values. However, on the basis of MCC values, the prediction accuracy of MPNN_sep was lower compared to SVM. Thus, MPNN representation learning clearly benefitted from the use of individual input graph components (see “Methods section”).

An important result of global performance comparison was that AC prediction accuracy did not scale with increasing methodological complexity. Although differences in median prediction accuracy between best-performing methods were small, SVM represented an overall preferred approach.

**Fig. 2:**
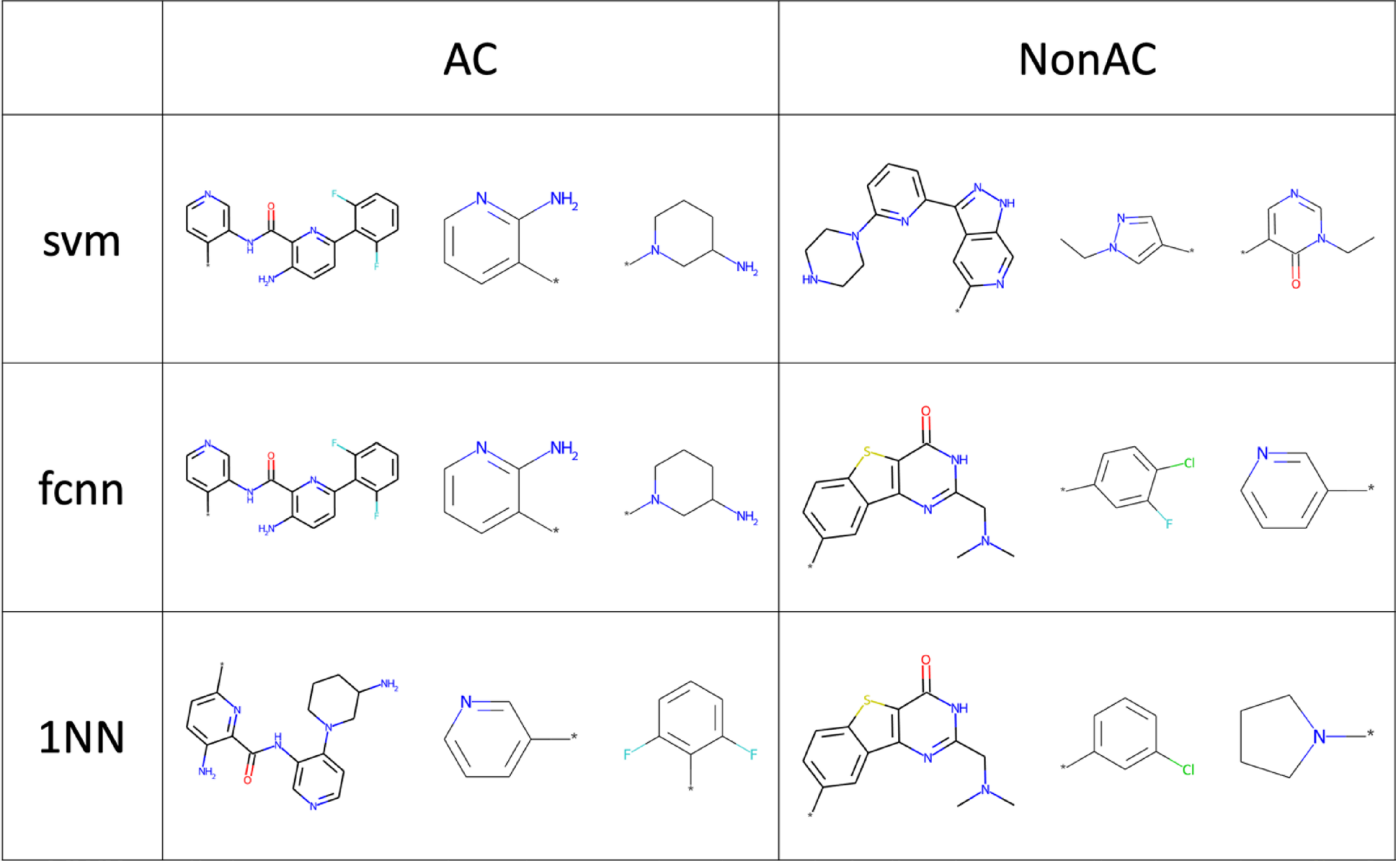
Correctly predicted test instances. Shown are exemplary ACs and nonACs that were correctly predicted using different methods. For each AC and nonAC, the MMP core is shown on the left, followed by the two substituent fragments representing the chemical transformation

Figure 2 shows exemplary ACs and nonACs from activity class ChEMBL4523 (Additional file 1: Table S1) that were accurately predicted using different methods. In these exemplary cases, MMP cores of ACs and nonACs were distinct, but essentially conserved among ACs and nonACs, respectively, thus providing a rationale for consistently accurate predictions.

### Influence of training set size

**Fig. 3:**
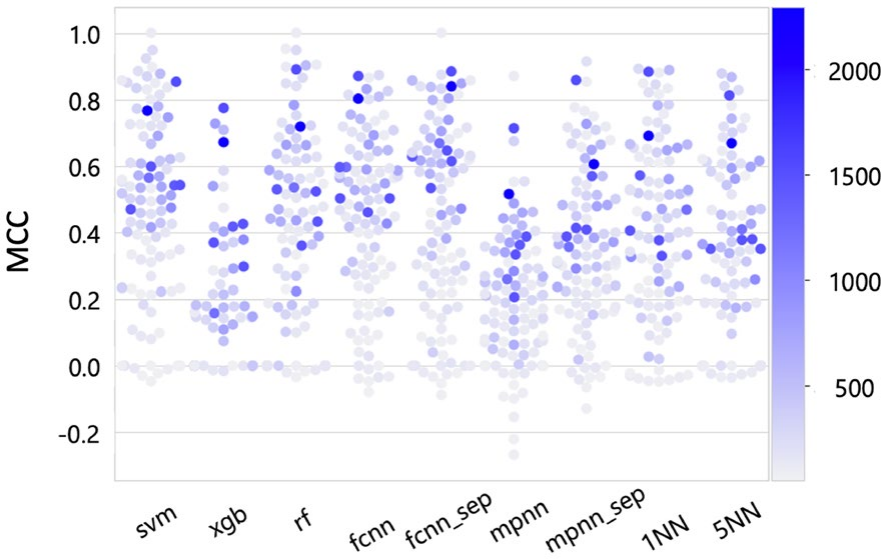
Influence of training set size on prediction accuracy. The swarm plot shows prediction accuracy on the basis of MCC values achieved by the different methods for training sets of varying size. Each of the 100 activity classes is represented by a dot that is color-coded according to the spectrum on the right according to the total number of MMPs in training sets (i.e., the darker the blue color, the larger the training set)

Given the different numbers of compounds comprising 100 activity classes (Additional file 1: Table S1), training sets for ML also varied in size. Therefore, we analyzed if there was a relationship between increasing training set sizes and prediction accuracy achieved by different methods. Especially for FCNN and MPNN, increasing prediction accuracy might be expected for increasingly large training sets. Figure 3 shows the effects of training set size on prediction accuracy.

There was no significant correlation between training set sizes and prediction accuracy for the different methods including MPNN and FCNN. Models yielding poor predictions were typically derived from small (or smallest) training sets and models based on large training sets generally achieved higher accuracy. However, best prediction accuracies for different methods were obtained on the basis of variably sized training sets including many small sets. Hence, compound class-specific differences affected predictions more than available training data volumes, which was consistently observed for ML methods of different complexity; another interesting finding.

### Data leakage phenomena

**Fig. 4:**
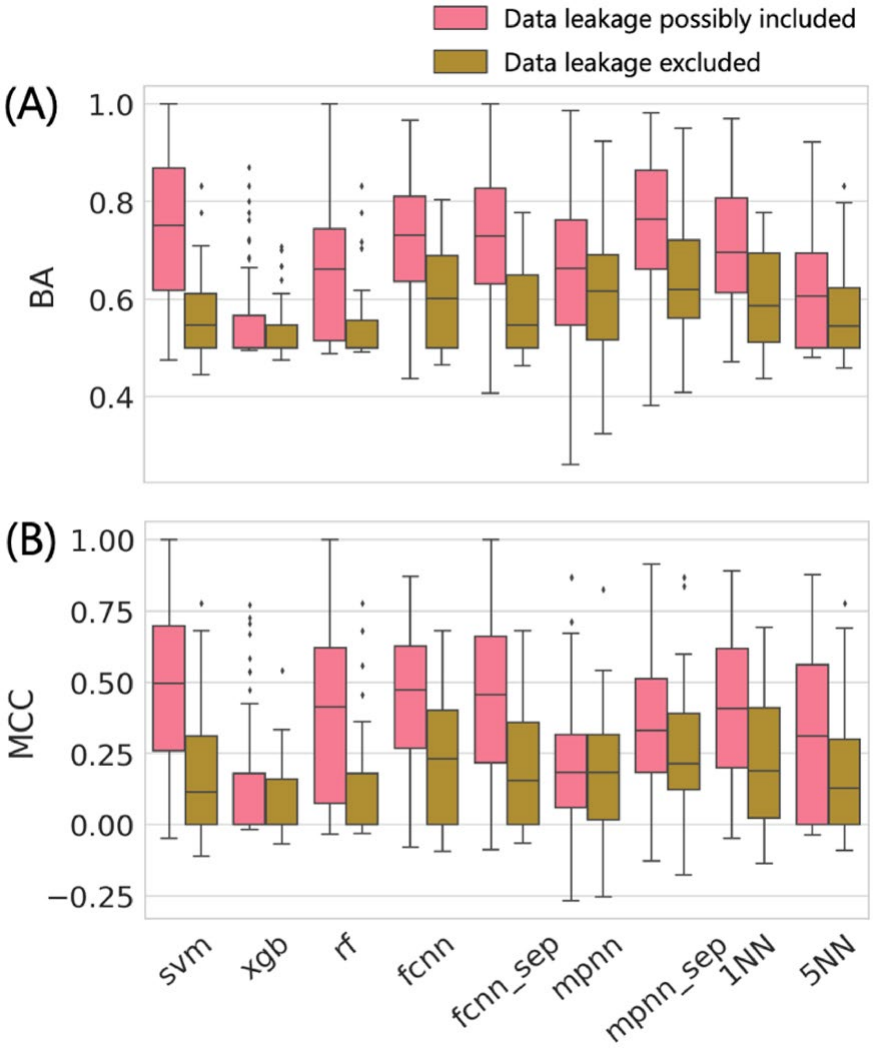
Influence of data leakage on prediction accuracy. Boxplots report the distribution of AC prediction accuracy for nine ML approaches across 42 activity classes on the basis of **A** BA and **B** MCC values according to Fig. 1 in the presence (pink boxes) or absence (brown) of data leakage (i.e., compound overlap between training and test sets)

We next investigated to which extent possible data leakage affected the predictions. In the context of AC predictions, data leakage corresponds to compound overlap between ACs or nonACs in training and test sets. In the absence of data leakage, ACs and nonACs in training and test sets are structurally distinct. Figure 4 reports the results of predictions in the presence and absence of data leakage. These predictions were carried out using 42 activity classes that were sufficiently large to yield meaningful training sets having no compound overlap with test sets. Global trends in prediction accuracy corresponded to those observed in Fig. 1. However, for all methods, prediction accuracy was significantly reduced if training and test sets were structurally distinct. Although most models were still predictive when data leakage was excluded, BA values were typically reduced to ~ 0.6 or less and MCC values to less than 0.25. Thus, compound overlap between MMPs used for training and testing had a strong positive effect on AC prediction accuracy, regardless of the methods that were used.

For ACs, compound overlap predominantly leads to memorization of highly potent compounds in ML because a highly potent compound can form ACs with multiple weakly potent analogues. In addition, for nonACs, many weakly potent compounds can be memorized. Clearly, predictions at the level of compound pairs such as ACs can be strongly supported by compounds shared in training and test pairs and the ensuing memorization effects. For structurally distinct training and test sets, pair-based prediction becomes much more challenging. The results in Fig. 4 also imply that generally high prediction accuracy obtained for ACs in independent studies (see “Introduction section”) was most likely supported by data leakage phenomena, providing a plausible explanation for the partly surprising success in addressing the principally challenging AC prediction task.

### Balanced versus imbalanced training sets

**Fig. 5:**
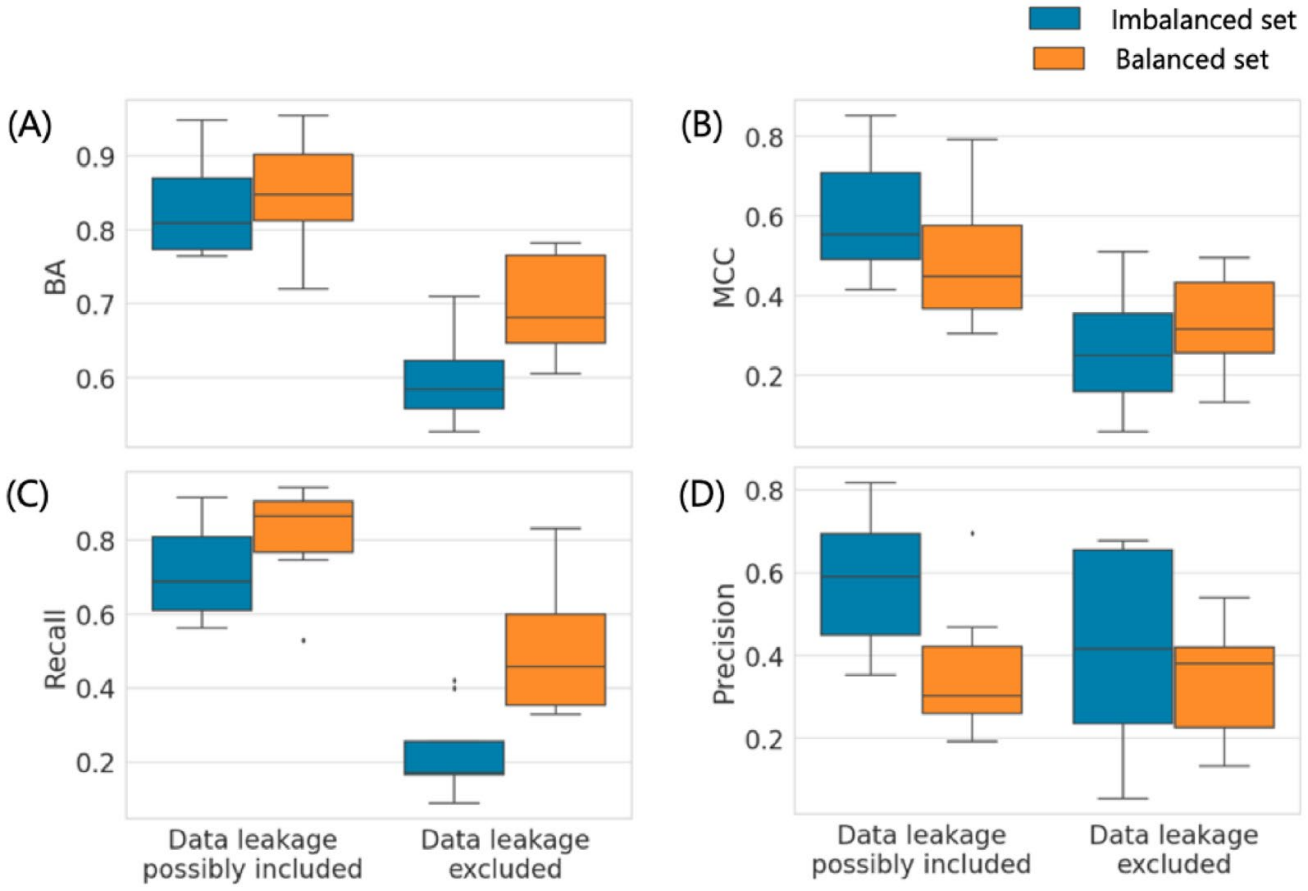
Balanced vs. imbalanced training sets for SVM models. Boxplots report the prediction accuracy of SVM models derived from imbalanced or balanced training sets of 10 activity classes with largest numbers of ACs on the basis of **A** BA, **B** MCC, **C** recall, and **D** precision in the presence or absence of data leakage

**Fig. 6:**
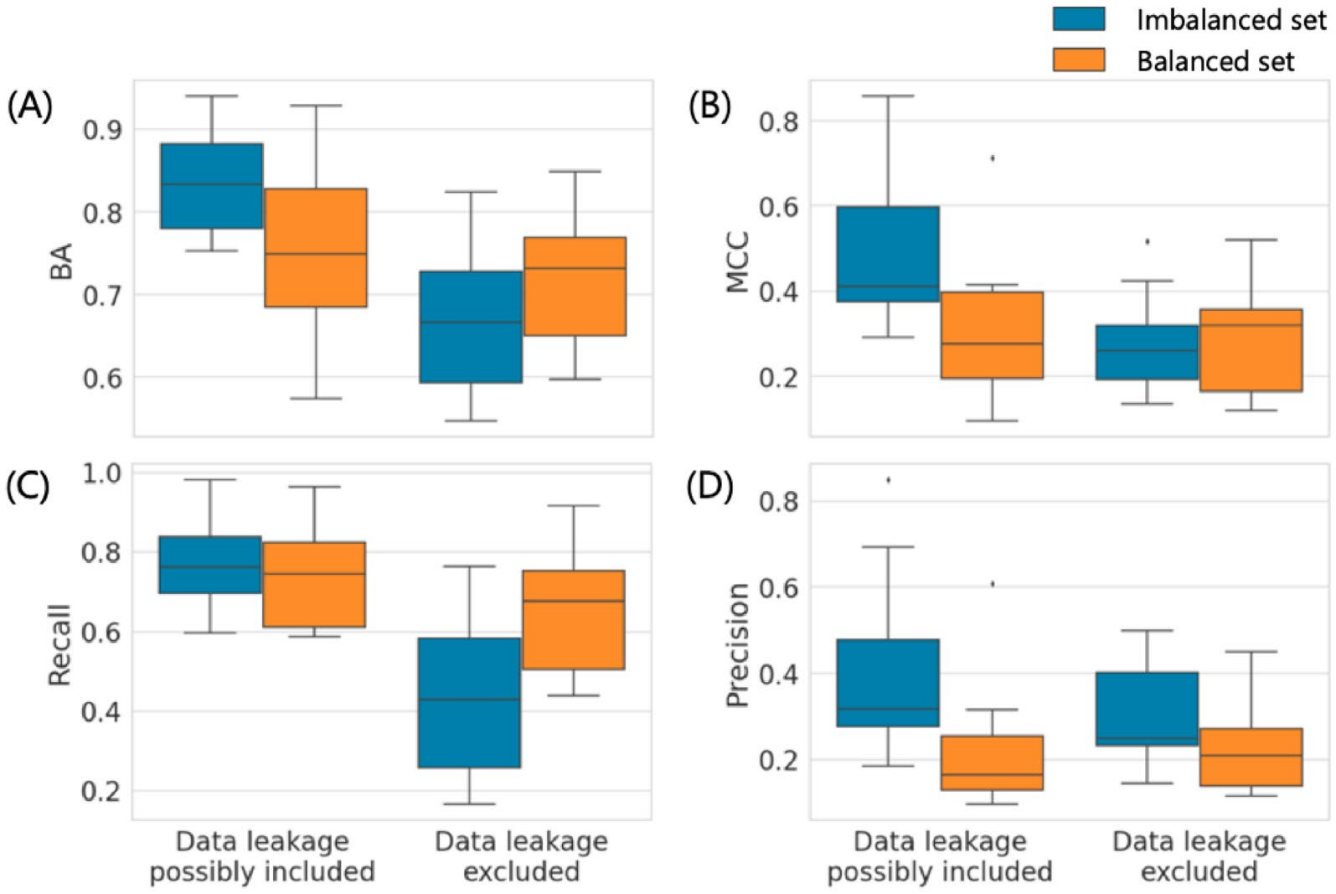
Balanced vs. imbalanced training sets for MPNNs. Boxplots report the prediction accuracy of MPNN_sep models derived from imbalanced or balanced training sets of 10 activity classes with largest numbers of ACs on the basis of **A** BA, **B** MCC, **C** recall, and **D** precision in the presence or absence of data leakage

Another issue of general relevance for ML concerns the preferred use of training sets with balanced class label composition. However, for AC predictions, imbalanced training sets provide a realistic application scenario because ACs are only rarely observed compared to nonACs, as discussed above. Nonetheless, we also investigated the influence of balanced training sets on AC predictions in the context of data leakage assessment. Therefore, the 10 activity classes containing the largest number of ACs were selected and the number of nonACs used for training was reduced to match the number of ACs to provide balanced learning conditions. Then, SVM and MPNN_sep models were derived on the basis of original (imbalanced) and balanced training sets, both in the presence or absence of data leakage, and the predictions were compared, as reported in Figs. 5 and 6, respectively. Here, prediction accuracy was assessed on the basis of BA and MCC values as well as recall and precision. For imbalanced training sets, overall prediction accuracy might be overestimated on the basis of some performance measures if the majority class (here nonACs) is more accurately predicted than the minority class (ACs). In such cases, MCC is becoming particularly relevant as a performance measure because it equally weighs TP, FN, TN, and FP .

Under varying calculation conditions considering both data balance and leakage, prediction characteristics changed in different ways. For SVM, BA and recall further increased for balanced relative to imbalanced training sets, both in the presence and absence of data leakage (Fig. 5A and C). However, on the basis of MCC, this performance increase was only observed when data leakage was excluded. In the presence of data leakage, the use of balanced training sets reduced MCC-based prediction accuracy compared to the original sets (Fig. 5B). Furthermore, precision was reduced for balanced relative to imbalanced training sets, both in the presence and absence of data leakage. Moreover, for MPNN_sep, the use of balanced compared to original training sets led to a decrease in BA in the absence and to an increase in the presence of data leakage (Fig. 6A). On the basis of MCC, prediction accuracy decreased for balanced sets in the presence and increased in the absence of data leakage (Fig. 6B), consistent with the observations made for SVM (Fig. 5B). In addition, precision also consistently decreased for balanced training sets (Figs. 5C and 6D), while recall of balanced MPNN_sep models only increased if data leakage was excluded (Fig. 6C).

Taken together, these results showed that for both methods, relative model performance based on imbalanced vs. balanced training sets depended on the presence or absence of data leakage. Furthermore, recall/precision characteristics differed from prediction accuracy trends depending on data balance and leakage conditions. Clearly, when compound overlap between training and test sets was permitted, MCC decreased when training sets were balanced, due to the reduction of the majority class, while an increase in MCC as a consequence of data balance was only observed when training and test sets were structurally distinct, reflecting an intricate interplay between these learning conditions in AC prediction.

## Conclusion

In this work, we have investigated AC predictions on a much larger scale than has been done before and with a particular focus on comparing a spectrum of ML methods of increasing complexity. In most cases, predictive models were obtained and prediction accuracy did not scale with ML model complexity. Even a simple 1NN classifier approached the accuracy level of overall best ML predictions obtained with SVM, FCNN, and MPNN_sep. The success of representation learning using MPNNs strongly depended on the graph input formats. However, the deep learning architectures investigated here did not provide an advantage over SVM that was the overall preferred approach across 100 activity classes (albeit by relatively small margins). By contrast, decision tree methods were overall less predictive. In particular, XGB that is extensively used in compound classification, displayed only poor performance in AC prediction. We also demonstrated that training set size was not a critical factor for AC prediction accuracy, perhaps surprisingly. For all models including deep neural networks, best predictions were often obtained on the basis of relatively small training sets, depending on individual activity classes. However, given that AC predictions depend on compound pairs, compound overlap between different ACs in training and test sets was shown to strongly support accurate predictions. For structurally distinct training and test sets, prediction accuracy was significantly reduced, as one might anticipate, yielding a more realistic assessment of AC predictions. We also observed an intricate interplay between varying data balance and leakage conditions on model performance, yielding different prediction characteristics and trends on the basis of alternative performance measures.

## Supplementary Information

**Additional file 1: Table S1.** Compound activity classes. **Table S2.** Hyperparameters of machine learning models. **Table S3.** Initial feature settings for atoms and bonds in message passing neural networks.

## Data Availability

All calculations were carried out with open source software as specified, except the OpenEye toolkit, for which a free academic license is required. Activity class data and calculation scripts used herein are freely available from https://github.com/tamshun/Large-Scale_ACPrediction.git.

## Abbreviations

AC: Activity cliff
AUC: Area under the curve
AXV: Advanced cross-validation
BA: Balanced accuracy
CGR: Condensed graph of reaction
DL: Deep learning
ECFP: Extended connectivity fingerprints
FCNN: Fully connected neural network
FN: False negative
FP: False positive
GNN: Graph neural network
MCC: Matthew’s correlation coefficient
MMP: Matched molecular pair
ML: Machine learning
MPNN: Message passing neural network
NN: Nearest neighbor
QSAR: Quantitative structure–activity relationship
ReLU: Rectified linear unit
RF: Random forest
ROC: Receiver operating characteristic
SAR: Structure–activity relationship
SVM: Support vector machine
TN: True negative
TP: True positive
XGB: Extreme gradient boosting
