# Supplementary material for: Large-scale prediction of activity cliffs using machine and deep learning methods of increasing complexity
Source: J Cheminform. 2023 Jan 7;15:4. doi: 10.1186/s13321-022-00676-7 (PMC9825040; doi:10.1186/s13321-022-00676-7)
Supplement: Supplementary file 1 — Additional file 1: Table S1. Compound activity classes. Table S2. Hyperparameters of machine learning models. Table S3. Initial feature settings for atoms and bonds in message passing neural networks. [file 13321_2022_676_MOESM1_ESM.docx]

**Additional file**

**Large-Scale Prediction of Activity Cliffs Using Machine and Deep Learning Methods of Increasing Complexity**

Shunsuke Tamura, Tomoyuki Miyao, and Jürgen Bajorath

**Tables S1–S3**

Table S1. Compound activity classes.

| ChEMBL  Target ID | Activity class name | #cpds | #mmp | #mms | Data leakage excluded set |
| --- | --- | --- | --- | --- | --- |
| 244 | Coagulation factor X | 507 | 3283 | 213 | TRUE |
| 204 | Prothrombin | 336 | 2179 | 115 | TRUE |
| 3594 | Carbonic anhydrase 9 | 315 | 2108 | 278 | TRUE |
| 261 | Carbonic anhydrase 1 | 350 | 2091 | 304 | TRUE |
| 205 | Carbonic anhydrase 2 | 347 | 2091 | 309 | TRUE |
| 264 | Histamine H3 receptor | 243 | 2017 | 134 | TRUE |
| 3242 | Carbonic anhydrase 12 | 289 | 1751 | 246 | TRUE |
| 217 | D(2) dopamine receptor | 211 | 1362 | 126 | TRUE |
| 253 | Cannabinoid receptor 2 | 257 | 1125 | 103 | TRUE |
| 3837 | Procathepsin L | 214 | 1102 | 69 | TRUE |
| 236 | Delta-type opioid receptor | 186 | 1093 | 44 | TRUE |
| 234 | D(3) dopamine receptor | 163 | 1054 | 98 | TRUE |
| 237 | Kappa-type opioid receptor | 190 | 1039 | 48 | TRUE |
| 251 | Adenosine receptor A2a | 169 | 1010 | 86 | TRUE |
| 228 | Sodium-dependent serotonin transporter | 154 | 923 | 78 | TRUE |
| 3880 | Heat shock protein HSP 90-alpha | 120 | 894 | 25 | TRUE |
| 284 | Dipeptidyl peptidase 4 | 146 | 884 | 47 | TRUE |
| 255 | Adenosine receptor A2b | 100 | 841 | 57 | TRUE |
| 256 | Adenosine receptor A3 | 104 | 796 | 65 | TRUE |
| 3991 | Coagulation factor VII | 89 | 775 | 24 | TRUE |
| 214 | 5-hydroxytryptamine receptor 1A | 156 | 708 | 108 | TRUE |
| 344 | Melanin-concentrating hormone receptor 1 | 133 | 686 | 62 | TRUE |
| 1075323 | Endoplasmin | 55 | 672 | 4 | TRUE |
| 2326 | Carbonic anhydrase 7 | 104 | 627 | 51 | TRUE |
| 233 | Mu-type opioid receptor | 160 | 625 | 46 | TRUE |
| 224 | 5-hydroxytryptamine receptor 2A | 144 | 608 | 101 | TRUE |
| 4657 | Dipeptidyl peptidase 8 | 66 | 601 | 8 | TRUE |
| 218 | Cannabinoid receptor 1 | 182 | 583 | 71 | TRUE |
| 2034 | Glucocorticoid receptor | 119 | 554 | 58 | TRUE |
| 267 | Proto-oncogene tyrosine-protein kinase Src | 78 | 501 | 72 | TRUE |
| 4793 | Dipeptidyl peptidase 9 | 63 | 500 | 8 | FALSE |
| 1902 | Peptidyl-prolyl cis-trans isomerase FKBP1A | 76 | 463 | 11 | TRUE |
| 2147 | Serine/threonine-protein kinase pim-1 | 103 | 459 | 40 | TRUE |
| 2820 | Coagulation factor XI | 105 | 436 | 24 | TRUE |
| 4409 | cAMP and cAMP-inhibited cGMP 3',5'-cyclic phosphodiesterase 10A | 79 | 422 | 16 | TRUE |
| 222 | Sodium-dependent noradrenaline transporter | 98 | 406 | 40 | TRUE |
| 229 | Alpha-1A adrenergic receptor | 99 | 391 | 36 | FALSE |
| 3729 | Carbonic anhydrase 4 | 91 | 391 | 50 | TRUE |
| 4523 | Serine/threonine-protein kinase pim-2 | 92 | 369 | 39 | TRUE |
| 1800 | Corticotropin-releasing factor receptor 1 | 82 | 361 | 34 | TRUE |
| 1862 | Tyrosine-protein kinase ABL1 | 62 | 355 | 43 | TRUE |
| 3717 | Hepatocyte growth factor receptor | 93 | 343 | 20 | FALSE |
| 226 | Adenosine receptor A1 | 84 | 320 | 44 | FALSE |
| 3371 | 5-hydroxytryptamine receptor 6 | 79 | 317 | 30 | TRUE |
| 4308 | B1 bradykinin receptor | 61 | 311 | 13 | FALSE |
| 2954 | Cathepsin S | 117 | 306 | 53 | FALSE |
| 219 | D(4) dopamine receptor | 61 | 302 | 40 | FALSE |
| 4072 | Cathepsin B | 108 | 287 | 43 | TRUE |
| 232 | Alpha-1B adrenergic receptor | 56 | 284 | 15 | FALSE |
| 335 | Tyrosine-protein phosphatase non-receptor type 1 | 78 | 278 | 23 | TRUE |
| 1871 | Androgen receptor | 63 | 268 | 23 | FALSE |
| 225 | 5-hydroxytryptamine receptor 2C | 91 | 264 | 54 | FALSE |
| 3759 | Histamine H4 receptor | 58 | 258 | 45 | FALSE |
| 3105 | Poly [ADP-ribose] polymerase 1 | 58 | 248 | 17 | FALSE |
| 259 | Melanocortin receptor 4 | 58 | 248 | 27 | FALSE |
| 238 | Sodium-dependent dopamine transporter | 89 | 234 | 32 | FALSE |
| 1855 | Gonadotropin-releasing hormone receptor | 65 | 231 | 21 | FALSE |
| 260 | Mitogen-activated protein kinase 14 | 64 | 224 | 18 | FALSE |
| 3891 | Calpain-1 catalytic subunit | 33 | 214 | 7 | FALSE |
| 2000 | Plasma kallikrein | 39 | 211 | 9 | FALSE |
| 1875 | 5-hydroxytryptamine receptor 4 | 48 | 208 | 8 | FALSE |
| 2842 | Serine/threonine-protein kinase mTOR | 60 | 206 | 41 | FALSE |
| 4822 | Beta-secretase 1 | 80 | 202 | 19 | FALSE |
| 280 | Collagenase 3 | 47 | 201 | 23 | FALSE |
| 3473 | C-C chemokine receptor type 3 | 33 | 200 | 35 | FALSE |
| 3510 | Carbonic anhydrase 14 | 60 | 199 | 30 | FALSE |
| 2617 | Tryptase alpha/beta-1 | 54 | 196 | 13 | FALSE |
| 6136 | Lysine-specific histone demethylase 1A | 59 | 183 | 17 | FALSE |
| 2360 | Hypoxanthine-guanine phosphoribosyltransferase | 23 | 179 | 14 | FALSE |
| 1833 | 5-hydroxytryptamine receptor 2B | 69 | 173 | 33 | FALSE |
| 1983 | 5-hydroxytryptamine receptor 1D | 71 | 169 | 29 | FALSE |
| 1741186 | Nuclear receptor ROR-gamma | 36 | 168 | 7 | FALSE |
| 1926488 | Protoporphyrinogen oxidase | 23 | 166 | 16 | FALSE |
| 2971 | Tyrosine-protein kinase JAK2 | 57 | 160 | 20 | FALSE |
| 4792 | Orexin receptor type 2 | 74 | 159 | 21 | FALSE |
| 4005 | Phosphatidylinositol 4,5-bisphosphate 3-kinase catalytic subunit alpha isoform | 68 | 155 | 40 | FALSE |
| 5023 | E3 ubiquitin-protein ligase Mdm2 | 49 | 151 | 20 | FALSE |
| 1898 | 5-hydroxytryptamine receptor 1B | 70 | 148 | 29 | FALSE |
| 333 | 72 kDa type IV collagenase | 48 | 144 | 14 | FALSE |
| 5407 | Serine/threonine-protein kinase pim-3 | 64 | 142 | 25 | FALSE |
| 1163125 | Bromodomain-containing protein 4 | 63 | 141 | 25 | FALSE |
| 216 | Muscarinic acetylcholine receptor M1 | 36 | 141 | 9 | FALSE |
| 5113 | Orexin/Hypocretin receptor type 1 | 64 | 139 | 19 | FALSE |
| 3018 | Suppressor of tumorigenicity 14 protein | 55 | 135 | 16 | FALSE |
| 4482 | Serine/threonine-protein kinase PAK 4 | 33 | 129 | 11 | FALSE |
| 1795117 | Histone-lysine N-methyltransferase, H3 lysine-79 specific | 36 | 129 | 6 | FALSE |
| 202 | Dihydrofolate reductase | 42 | 121 | 35 | FALSE |
| 235 | Peroxisome proliferator-activated receptor gamma | 38 | 119 | 11 | FALSE |
| 3922 | Methionine aminopeptidase 2 | 11 | 117 | 8 | FALSE |
| 2079846 | Son of sevenless homolog 1 | 29 | 114 | 3 | FALSE |
| 2056 | D(1A) dopamine receptor | 37 | 113 | 24 | FALSE |
| 3267 | Phosphatidylinositol 4,5-bisphosphate 3-kinase catalytic subunit gamma isoform | 47 | 98 | 15 | FALSE |
| 321 | Matrix metalloproteinase-9 | 49 | 95 | 20 | FALSE |
| 4361 | Induced myeloid leukemia cell differentiation protein Mcl-1 | 34 | 90 | 18 | FALSE |
| 1946 | Melatonin receptor type 1B | 6 | 88 | 7 | FALSE |
| 2014 | Nociceptin receptor | 28 | 86 | 8 | FALSE |
| 3979 | Peroxisome proliferator-activated receptor delta | 34 | 74 | 6 | FALSE |
| 208 | Progesterone receptor | 53 | 73 | 21 | FALSE |
| 203 | Epidermal growth factor receptor | 32 | 69 | 12 | FALSE |
| 262 | Glycogen synthase kinase-3 beta | 35 | 68 | 22 | FALSE |

#### For each activity class, the ChEMBL target ID, number of compounds used for MMP generation (#cpd), number of generated MMPs (#mmp), number of matching molecular series (#mms) which consist of all MMPs having a common core structure, and Boolean values indicating whether activity classes were part of the “data leakage excluded set” or not are provided. The activity classes are ordered by the number of MMPs.

Table S2. Hyperparameters of machine learning models.

| Method | Hyperparameter | Range(interval) | log scale |
| --- | --- | --- | --- |
| rf | number of decision trees | 50 - 1000 (1) | False |
|  | maximum depth of a tree | 4 - 50 (1) | False |
|  | minimum samples to split a node | 10^-8^ - 1.0 | True |
| xgb | alpha for L1 regulation | 10^-8^ - 1.0 | True |
|  | lambda for L2 regulation | 10^-8^ - 1.0 | True |
|  | subsample ratio | 0.2 - 1.0 | False |
|  | colsample rate by tree | 0.2 - 1.0 | False |
|  | maximum depth of a tree | 3 - 9 (2) | False |
|  | minimum sum of weights in split nodes | 2 - 10 (1) | False |
|  | eta | 10^-8^ - 1.0 | True |
|  | gamma | 10^-8^ - 1.0 | True |
| fcnn | number of hidden layers | 2 - 3 (1) | False |
|  | dropout rate | 0.25 - 0.50 (0.25) | False |
|  | learning rate of adam optimizer | 10^-4^ - 10^-2^ | False |
|  | number of steps for the scheduler | 1 - 3 (1) | False |
| fcnn_sep | number of hidden layers for core | 1 - 4 (1) | False |
|  | number of hidden layers for substituents | 1 - 4 (1) | False |
|  | number of hidden layers for concatenated feature vector | 2 - 8 (1) | False |
|  | dimension of feature vector for core | 70 - 100 (10) | False |
|  | dimension of feature vector for substituents | 70 - 100 (10) | False |
|  | dropout rate | 0.1 - 0.3 (0.05) | False |
|  | learning rate of adam optimizer | 10^-4^ - 10^-2^ | False |
|  | number of steps for the scheduler | 1 - 3 (1) | False |
| mpnn | number of convolutions | 2 - 4 (1) | False |
|  | dimension of feature vector | 70 - 100 (10) | False |
|  | number of hidden layers of FCNN | 2 - 8 (1) | False |
|  | dropout rate | 0.1 - 0.3 (0.05) | False |
|  | learning rate of adam optimizer | 10^-4^ - 10^-2^ | False |
|  | number of steps for the scheduler | 1 - 3 (1) | False |
| mpnn_sep | number of convolutions for core | 2 - 4 (1) | False |
|  | number of convolutions for substituents | 2 - 4 (1) | False |
|  | dimension of feature vector for core | 70 - 100 (10) | False |
|  | dimension of feature vector for substituents | 70 - 100 (10) | False |
|  | number of hidden layers of FCNN | 2 - 8 (1) | False |
|  | dropout rate | 0.1 - 0.3 (0.05) | False |
|  | learning rate of adam optimizer | 10^-4^ - 10^-2^ | False |
|  | number of steps for the scheduler | 1 - 3 (1) | False |

Table S3. Initial feature settings for atoms and bonds in message passing neural networks.

| Part | Attribute | Descriptor | Dimensions |
| --- | --- | --- | --- |
| Atom | Atom type | Atomic number | 118 |
|  | Degree | Number of neighboring heavy atoms | 6 |
|  | Formal Charge | Formal charge (− 2, − 1, 0, 1, 2) | 5 |
|  | Chirality label | Nothing, R, S, or unrecognized | 4 |
|  | Hybridization | sp, sp^2^ , sp^3^ , sp^3^ d, or sp^3^ d^2^ | 5 |
|  | Aromaticity | Aromatic or not aromatic | 1 |
| Bond | Bond type | Single, double, triple, or aromatic ring | 4 |
|  | Ring | Ring bond or non-ring bond | 1 |
|  | Bond stereo | None, any, Z, E, cis, or trans | 6 |
